# Evaluation of myocardial function by strain echocardiography in cats with hypertrophic cardiomyopathy phenotypes

**DOI:** 10.14202/vetworld.2026.3027-3038

**Published:** 2026-07-17

**Authors:** Xufeng Ying, Chattida Panprom, Soontaree Petchdee

**Affiliations:** 1 Graduate School, Science and Innovation for Animal Health Program, Faculty of Veterinary Medicine, Kasetsart University, Thailand.; 2 Department of Large Animal and Wildlife Clinical Sciences, Faculty of Veterinary Medicine, Kasetsart University, Kamphaeng Saen, Nakhon Pathom, Thailand.

**Keywords:** cardiomyopathy, cats, echocardiography, left atrial reservoir strain, myocardial strain, right ventricular longitudinal strain, speckle-tracking echocardiography, ventricular function

## Abstract

**Background and Aim:**

Hypertrophic cardiomyopathy (HCM) is the most common cardiac disease in cats, and early detection of myocardial dysfunction remains challenging using conventional echocardiography alone. This study evaluated myocardial function using two-dimensional speckle-tracking echocardiography and investigated the diagnostic value of left ventricular global longitudinal strain (LVGLS), right ventricular longitudinal strain (RVLS), left atrial reservoir strain (LARS), and strain–volume curve analysis for differentiating HCM phenotypes in cats.

**Materials and Methods:**

A retrospective observational study was conducted on 40 client-owned cats categorized into three groups: Healthy controls (n = 15), cats with a hypertrophic phenotype and normal left atrial size (n = 13), and cats with a hypertrophic phenotype accompanied by left atrial enlargement (n = 12). Comprehensive echocardiographic examinations included conventional measurements together with LVGLS, RVLS, LARS, circumferential strain, and strain–volume curve analysis. Group comparisons and correlations between strain-derived and conventional echocardiographic parameters were analyzed using appropriate statistical methods, with significance set at p < 0.05.

**Results:**

Cats with HCM and left atrial enlargement exhibited significantly lower LVGLS and LARS than healthy cats, indicating impaired ventricular systolic deformation and reduced atrial reservoir function. RVLS did not differ significantly among groups, suggesting relative preservation of right ventricular systolic function during the early stages of disease. Strain–volume curve analysis demonstrated progressive flattening and rightward displacement in affected cats, reflecting impaired myocardial deformation despite preserved conventional systolic indices such as fractional shortening. Moderate correlations were observed between LVGLS and radial strain, whereas LARS showed inverse correlations with indices of left ventricular hypertrophy. These findings indicate that strain-derived parameters provide complementary functional information beyond conventional echocardiographic measurements.

**Conclusion:**

Multi-chamber strain echocardiography integrating LVGLS, RVLS, LARS, and strain–volume curve analysis provides a comprehensive assessment of myocardial mechanics in cats with HCM. This approach improves characterization of hypertrophic phenotypes, facilitates earlier recognition of myocardial dysfunction, and may enhance disease staging and clinical decision-making in feline cardiology.

## INTRODUCTION

Feline cardiomyopathy is a significant cause of morbidity and mortality in cats, with hypertrophic cardiomyopathy (HCM) being the most prevalent form [1, 2]. Conventional echocardiography remains the cornerstone of diagnosis, but it often fails to detect early or subtle myocardial dysfunction [3, 4]. Strain echocardiography, which uses speckle-tracking methods, enables quantitative evaluation of myocardial deformation and has been shown to detect subclinical changes before overt structural alterations [5–9]. In addition to the left ventricular (LV), the right ventricular (RV) plays a critical role in overall cardiac performance. In feline HCM, secondary pulmonary hypertension or interventricular interactions may compromise RV function [10, 11]. Assessment of right ventricular longitudinal strain (RVLS) provides a sensitive and reproducible indicator of systolic function and may reflect global myocardial disease burden [12]. Left atrial (LA) function also provides prognostic information, as it reflects LV filling pressures and diastolic compliance [13, 14]. Among left atrial strain components, the left atrial reservoir strain (LARS) represents the reservoir function during ventricular systole and offers incremental diagnostic value beyond standard volumetric measures [15–17].

Strain echocardiography allows simultaneous evaluation of LV, RV, and LA deformation throughout the cardiac cycle, providing a comprehensive overview of myocardial mechanics [18]. Myocardial strain imaging can be assessed using either tissue Doppler imaging (TDI) or speckle-tracking echocardiography (STE). TDI-derived strains are limited by angle dependence and susceptibility to artifacts, making them less reliable in small-animal patients. In contrast, STE is angle-independent and more robust and is therefore the preferred method in both research and clinical practice. STE evaluates myocardial deformation by tracking natural acoustic markers within the myocardium on two-dimensional echocardiographic images [19, 20]. These speckles are automatically identified and followed frame by frame throughout the cardiac cycle. The relative displacement of speckles provides quantitative information on myocardial shortening and lengthening, which is expressed as strain. Left ventricular global longitudinal strain (LVGLS) is a sensitive marker of early systolic dysfunction, whereas RVLS primarily reflects free-wall contractility. Strain echocardiography is strongly correlated with disease severity in feline and canine cardiomyopathies [21–23]. Accurate assessment of LV diastolic function and filling pressures remains challenging in cats due to the effects of preload and heart rate on traditional Doppler parameters (E/A ratio and tricuspid regurgitation velocity) [24]. LARS, measured by STE, quantifies atrial compliance and reservoir capacity and serves as a functional marker of LV diastolic performance [25, 26]. Reduced LARS was associated with increased LV wall thickness, impaired LV function, and a higher LA/Aorta (AO) ratio, which is consistent with impaired LV relaxation and elevated filling pressures. Studies in humans have shown that decreased LARS is associated with LV stiffness and diastolic dysfunction [27]. The incorporation of strain imaging into feline echocardiographic evaluation may enable early detection of diastolic impairment before LA enlargement develops [28]. Most feline studies have evaluated LV or LA strain independently, whereas integrated assessment of LV, RV, and LA deformation remains limited. Rather than serving as primary diagnostic tools, strain-derived indices may provide complementary functional information beyond conventional two-dimensional echocardiography, particularly in cats with borderline structural changes or early disease stages (B1–B2). Identifying phenotype- and stage-related differences in myocardial deformation may improve understanding of disease pathophysiology and support future investigations into their prognostic value.

Although conventional echocardiography remains the cornerstone of HCM diagnosis in cats, it often fails to detect early or subtle myocardial dysfunction. Previous studies have evaluated LV or LA strain independently, with limited integration of multi-chamber assessment. There is a clear need for a comprehensive evaluation that combines LVGLS, RVLS, LARS, and strain–volume curve analysis to better characterize hypertrophic phenotypes, distinguish subclinical dysfunction, and improve disease staging. The integration of these advanced strain parameters may provide additional functional information beyond conventional indices such as fractional shortening and LA/AO ratio, particularly in cats with borderline structural changes or early disease stages. Furthermore, the relationship between these strain-derived indices and disease progression, as well as their prognostic value, remains insufficiently explored in feline cardiology.

Accordingly, this study aimed to assess myocardial function in cats using strain echocardiography and to determine the diagnostic utility of LVGLS, RVLS, and LARS for distinguishing different cardiomyopathic patterns. We hypothesized that LARS and multi-chamber strain parameters provide additional functional information beyond conventional indices such as LA/AO ratio and fractional shortening, and may facilitate earlier recognition of myocardial dysfunction in feline HCM.

## MATERIALS AND METHODS

### Ethical approval

The study protocol was reviewed and approved by the Animal Care and Use Committee of Kasetsart University, Thailand (approval no. ACKU-65-VET-077). This retrospective observational study used clinical and echocardiographic data obtained from client-owned cats presented to Kasetsart University Animal Teaching Hospital between May 2024 and May 2025. All procedures were performed as part of routine clinical cardiac evaluation and did not involve any experimental intervention beyond standard diagnostic care.

Written informed consent was obtained from the owners or legal custodians of all cats before clinical examination and use of anonymized medical data for research purposes. Patient confidentiality was maintained throughout the study by removing all owner-identifying information and using anonymized clinical records for data analysis. No animals or people are identifiable in this manuscript. All procedures were conducted in accordance with institutional animal ethics guidelines and accepted standards for retrospective veterinary clinical research.

### Study period and location

Echocardiographic examinations were performed between May 2024 and May 2025. Clinical data were obtained from the Kasetsart University Animal Teaching Hospital, Faculty of Veterinary Medicine, Kasetsart University, Kamphaeng Saen, Nakhon Pathom, Thailand.

### Study design and case enrollment

A retrospective observational study was conducted from May 2022 to May 2025. Forty client-owned cats were recruited for cardiac evaluation. The inclusion criteria were a sinus rhythm, fractional shortening greater than 25%, and the availability of complete echocardiographic datasets, including three-dimensional full-volume acquisitions. Cats with poor image quality and unclear speckle-tracking were excluded. Cats were classified into the following groups on the basis of their clinical history and echocardiographic findings: healthy controls (Group 1), characterized by the absence of clinical or echocardiographic evidence of heart disease; cardiomyopathy with a normal LA/AO ratio (Group 2), characterized by increased myocardial thickening without LA enlargement; and cardiomyopathy (Group 3), characterized by myocardial hypertrophy and LA dilation. Hypertrophic phenotype is defined as IVSd and/or LV posterior wall thickness at end-diastole (LVPWd) ≥6 mm in diastole and increased LA size with LA/AO >1.5. In addition, secondary causes of myocardial hypertrophy, such as hyperthyroidism and systemic hypertension, were excluded through clinical evaluation.

### Echocardiography

An echocardiographic examination was performed by an experienced cardiologist using a Vivid E95 ultrasound system (GE Healthcare, Horten, Norway) equipped with a 2–8 MHz phased-array transducer. Cats were scanned in right and left lateral recumbency without sedation. Standard two-dimensional, M-mode, and Doppler echocardiography were performed in accordance with American College of Veterinary Internal Medicine (ACVIM) guidelines [29]. Images for strain evaluation were obtained from the three apical views, ensuring adequate endocardial border definition. Images were acquired by an experienced operator and analyzed offline using vendor-provided software (EchoPac AFI; GE Healthcare, version 206).

### Strain and myocardial work analysis

Global longitudinal strain of the LV (LVGLS) was calculated from three apical planes. Circumferential, radial, right ventricular strain, and LA strain (reservoir) were also obtained. Strain curves were constructed by plotting strain values against end-diastolic and end-systolic volumes throughout the cardiac cycle. Strain–volume curves were generated by synchronizing speckle-tracking-derived strain values with left ventricular volume changes throughout the cardiac cycle, allowing visualization of deformation–volume relationships. The technique in the present study was adapted from human and canine cardiology. Myocardial work was integrated with the blood pressure values and global longitudinal strain (GLS) by time alignment. Echocardiographic acquisition parameters were applied at a frame rate (≥100 fps) for speckle-tracking analysis. The standardized imaging views were evaluated as follows, with manual contour adjustments made as needed to ensure tracking quality. LVGLS was evaluated from apical 4-, 2-, and 3-chamber views, RVLS was evaluated from an RV-focused apical 4-chamber view, and LARS was evaluated from an apical 4-chamber view optimized for LA. However, intra-observer variability analysis was reported as correlation coefficients.

### Statistical analysis

Continuous data were expressed as mean ± standard deviation (SD). Group comparisons were performed using one-way analysis of variance with Tukey post hoc analysis or Student’s *t*-test as appropriate. Correlations between strain parameters and left ventricular volumes were assessed with Spearman’s correlation coefficient. A p < 0.05 was considered statistically significant. Multiple comparisons were tested using the Bonferroni method, and the Shapiro–Wilk test was used to assess normality. Statistical analyses were conducted using GraphPad Prism 10 (GraphPad Software, San Diego, CA, USA).

## RESULTS

### The baseline characteristics of the study animals

In the present study, forty cats were included (22 males, 18 females), with a mean age of 4.91 ± 2.68 years. Scottish Fold (n = 13) and domestic short hair (n = 7) were the most frequently represented breeds, followed by Persian (n = 6). The remaining cats included Maine Coon (n = 5), British Shorthair (n = 3), and mixed breeds (n = 6). Because of the small number of cats in some breed categories, breed-based comparisons were not performed. Mean LV fractional shortening was 43.60 ± 5.9%. The enrolled animals consisted of 40 cats, divided into three groups: Healthy controls (Group 1, n = 15) with no clinical or echocardiographic evidence of heart disease. HCM phenotype with a normal LA/AO ratio (Group 2, n = 13), increased myocardial thickening without LA enlargement. Hypertrophic phenotype (Group 3, n = 12) with myocardial hypertrophy and LA dilation. The most prevalent breeds among cats were Scottish fold (n = 12; 30%), domestic shorthair (n = 7; 17.5%), Persian (n = 6; 15%), Maine Coon (n = 5; 12.5%), British shorthair (n = 3; 7.5%), mixed breeds (n = 6; 15%), and other breeds (n = 1; 2.5%). Other breeds had fewer than two cats. As reported in Table 1.

**Table 1:** Clinical characteristics of the study animals.

**Parameters**	**Group 1 (N = 15)**	**Group 2 (N = 13)**	**Group 3 (N = 12)**	**p-value**
Age (years)	5.06 ± 2.27	3.65 ± 1.89	6.08 ± 3.25	0.0768
Body weight (kg)	3.74 ± 1.22	4.46 ± 2.03	5.15 ± 1.10	0.1039
Male (number (%))	5/15 (33.33%)	6/13 (46.15%)	11/12 (91.66%)	-
Blood pressure (mmHg)	138.04 ± 15.34	128.34 ± 20.34	137.50 ± 5.88	0.410
Heart rate (bpm)	180.93 ± 31.35	183.00 ± 29.87	154.02 ± 31.21	0.0597

Data are presented as mean ± SD for continuous variables or as percentages for categorical variables. Indicated statistically significant difference (p < 0.05).

### Echocardiographic parameters of the study animals

The echocardiographic parameters of all cats are summarized in Table 2. There were no significant differences among groups in aortic diameter (AO), interventricular septal thickness in diastole (IVSd), LVPWd, left ventricular internal dimension in systole (LVIDs), or fractional shortening (FS) (p > 0.05 for all). In contrast, cats in Group 3 demonstrated significantly increased LA diameter, LA/AO ratio, and LA volume compared with healthy controls (Group 1) (p < 0.05), accompanied by a corresponding decrease in LVGLS (p < 0.0001). Furthermore, interventricular septal thickness in systole (IVSs) and left ventricular posterior wall thickness in systole (LVPWs) were markedly higher in Group 3 relative to Group 1 (p < 0.05), consistent with a hypertrophic myocardial phenotype.

### Strain analysis

**Figure 1:**
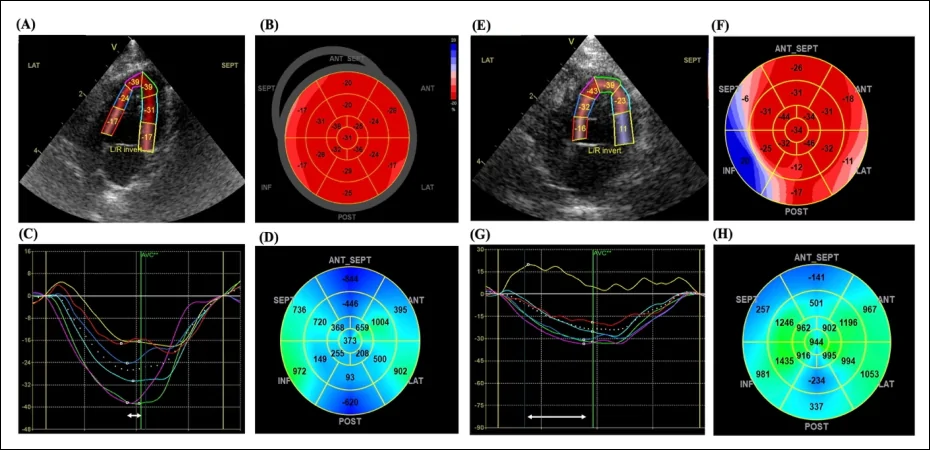
(A-D) Left ventricular images of a healthy cat and (E-H) a cat with HCM An image of the LV through the apical long-axis. The LV is traced, and speckles are placed throughout the myocardium. The movements of these speckles are tracked throughout the cardiac cycle. (A, C) Each colored line shows the amount of strain of a left ventricular segment. (B) The integrated strain is incorporated into a 17-segment model of the LV, representing global strain. (C) The strain curve. (D) The myocardial work. Figure E–H. Representative images of LS analysis in a cat with hypertrophic phenotype. (E) Apical long-axis view with segmental LS tracking. (F) Polar (bull’s-eye) projection of regional LS values showing segmental differences in deformation. (G) Strain–time curves from multiple segments, illustrating asynchronous contraction. (H) Quantitative strain dispersion map highlighting non-uniform myocardial function across LV regions.

**Figure 2:**
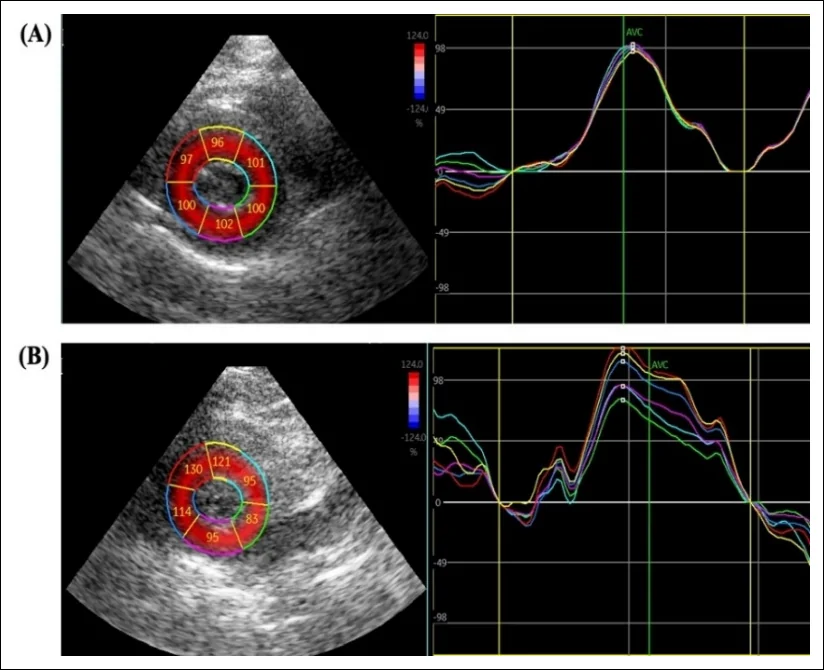
Mid-ventricular short-axis view depicting circumferential strain analysis in cats. (A) Representative example from a healthy cat showing homogeneous segmental contraction and uniform peak strain. (B) A cat with hypertrophic cardiomyopathy demonstrates reduced strain amplitude and temporal dispersion across myocardial segments, indicating early mechanical dyssynchrony.

**Figure 3:**
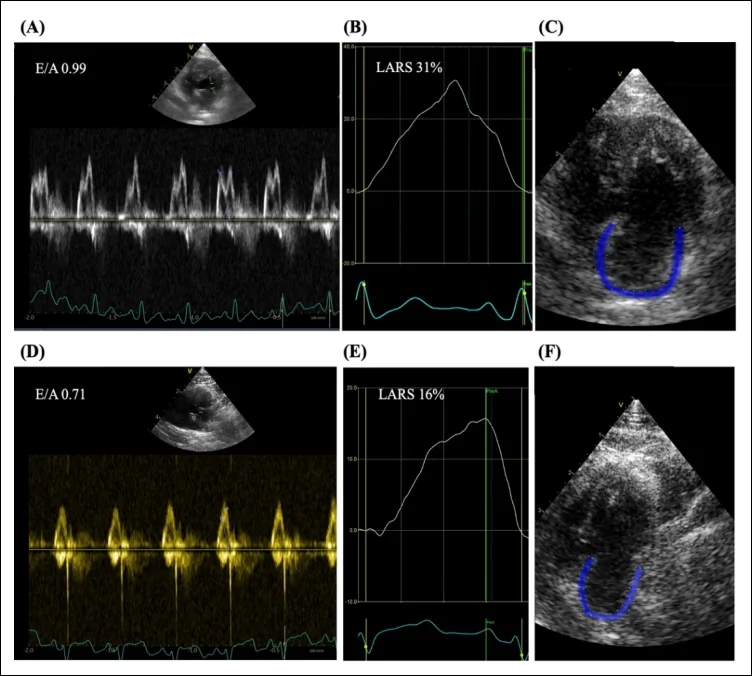
Representative echocardiographic assessment of left atrial (LA) strain in cats. (A–C) Echocardiographic images obtained from a healthy cat (Group 1). (A) Pulsed-wave Doppler mitral inflow profile. (B) Left atrial reservoir strain (LARS) curve. (C) Apical four-chamber view showing the color-coded region of interest used for two-dimensional speckle-tracking analysis of LA strain. (D–F) Representative echocardiographic findings from a cat with hypertrophic cardiomyopathy (Group 2). (D) Pulsed-wave Doppler mitral inflow demonstrating a reduced E/A ratio (0.71), consistent with impaired left ventricular relaxation (diastolic dysfunction). (E) LARS curve demonstrating reduced peak reservoir strain (16%), indicative of impaired left atrial compliance. (F) Two-dimensional speckle-tracking image illustrating the color-coded tracking of the LA wall (blue) used for LARS measurement.

**Figure 4:**
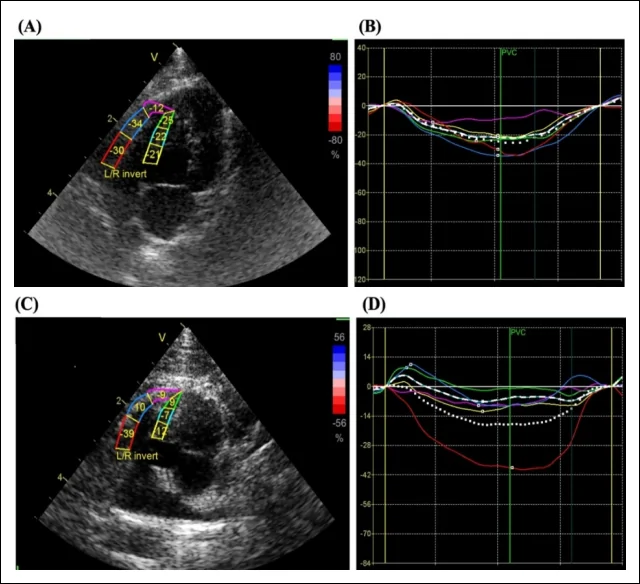
(A) A six-segment model of the endocardial borders in an RV for the free wall and the ventricular septum. (B) The global (dotted line) and free-wall RV (dashed line) LS curves of a cat in Group 1. Figure C-D. Right ventricular longitudinal strain analysis in a cat with hypertrophic cardiomyopathy. (C) RV-focused apical four-chamber view showing segmentation of the right ventricular free wall for strain analysis. (D) Strain-time curves depicting longitudinal deformation of individual RV segments. The basal segment demonstrates markedly reduced strain, indicating early regional RV dysfunction in Group 3.

Figures 1–4 show strain echocardiography in healthy cats and cats with HCM. Healthy cats (Group 1) exhibit steep, tightly clustered strain–volume relationships, indicating normal myocardial deformation and preserved ventricular–atrial coupling. Cats with hypertrophic phenotypes and left atrium enlargement (Group 3) show flatter, right-shifted strain–volume curves, consistent with reduced myocardial strain amplitude and progressive chamber dilatation. Figures 1D and 1H show a transition from higher, more uniform, and predominantly positive myocardial work in healthy cats to lower, heterogeneous, and partially negative myocardial work in cats with HCM.

The reference intervals for feline LVGLS are approximately –21% to –37%. Quantitatively, Group 3 cats exhibited significantly lower strain-curve slopes and ratios than controls (p < 0.05), indicating impaired relaxation despite preserved or mildly reduced chamber volumes. The flattened and right-shifted strain–volume curves in Group 3 cats, compared with steep and clustered patterns in healthy cats, indicate impaired deformation-volume coupling. RVLS remained relatively preserved across groups. The correlation between RVLS and LVGLS was modest and not statistically significant (r = 0.26, p = 0.17). This observation is now presented as a notable finding suggesting limited biventricular involvement in early or moderate disease stages.

### The Correlations between Echocardiographic Parameters

**Figure 5:**
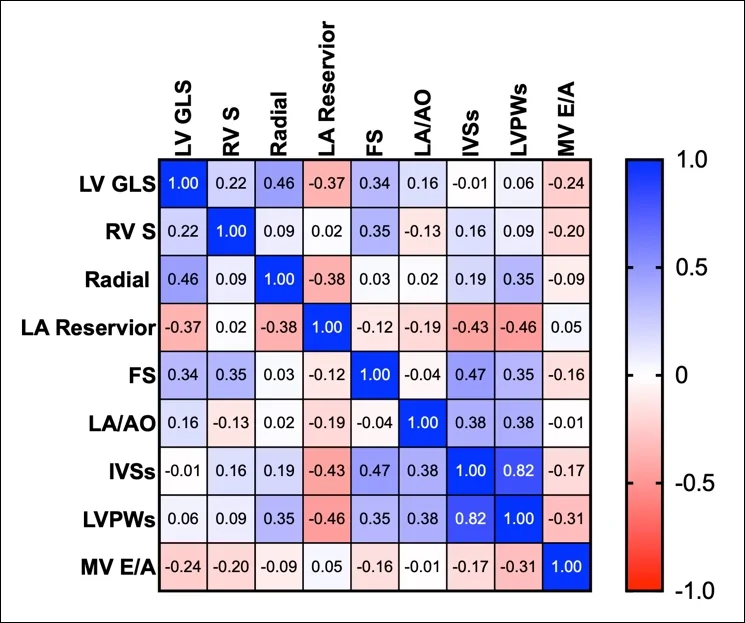
Spearman correlation heatmap between strain parameters and conventional echocardiographic indices in cats. Positive correlations are shown in blue, and negative correlations in red, with color intensity corresponding to correlation strength. Left ventricular global longitudinal strain (LVGLS) correlated moderately with radial strain and fractional shortening, while left atrial reservoir strain (LARS) demonstrated inverse associations with indices of wall thickness (IVSs and LVPWs). Weak relationships were observed between strain and the mitral inflow E/A ratio, indicating relative independence of strain from conventional diastolic measurements.

Spearman correlation analysis between strain parameters and conventional echocardiographic indices is presented in Figure 5. LVGLS showed a moderate positive correlation with radial strain (r = 0.46) and FS (r = 0.34), indicating that myocardial longitudinal deformation is moderately associated with circumferential and radial contractility as well as global systolic performance. Conversely, LVGLS demonstrated weak or negligible correlations with interventricular septal thickness in systole (IVSs), LVPWs, and mitral inflow E/A ratio (r = –0.01, 0.06, and –0.24, respectively), suggesting that strain-derived systolic indices are relatively independent of wall thickness and diastolic filling patterns. Right ventricular strain (RVS) correlated weakly with LVGLS (r = 0.22) and FS (r = 0.35), while LARS demonstrated mild to moderate inverse correlations with indices of left ventricular hypertrophy, including IVSs (r = –0.43) and LVPWs (r = –0.46). These findings imply that increasing wall thickness is associated with reduced atrial compliance and reservoir function. However, LARS did not correlate with conventional diastolic indices (E/A ratio, r = 0.05). Collectively, these correlations highlight the complementary relationship between deformation parameters and conventional echocardiographic measures, underscoring the utility of strain imaging in assessing subclinical myocardial and atrial dysfunction in feline cardiomyopathy.

**Table 2:** Echocardiographic parameters of the study animals.

**Parameters**	**Group 1 (N = 15)**	**Group 2 (N = 13)**	**Group 3 (N = 12)**	**p-value**
LA (cm)	1.17 ± 0.15*	1.19 ± 0.19	1.55 ± 0.55*	0.0223
LA/AO ratio	1.40 ± 0.12**	1.40 ± 0.09##	1.85 ± 0.48**##	0.0003
MPA/AO ratio	0.98 ± 0.04	1.0 ± 0.08	1.06 ± 0.22	0.4403
IVSd (cm)	0.47 ± 0.11	0.53 ± 0.15	0.61 ± 0.18	0.0688
LVPWd (cm)	0.49 ± 0.11	0.51 ± 0.10	0.59 ± 0.13	0.0591
LVIDd (cm)	1.35 ± 0.27	1.34 ± 0.23	1.52 ± 0.24	0.1617
IVSs (cm)	0.57 ± 0.11**	0.64 ± 0.17	0.78 ± 0.16**	0.0054
LVPWs (cm)	0.50 ± 0.11**	0.59 ± 0.15	0.69 ± 0.15**	0.0047
LVIDs (cm)	0.83 ± 0.23	0.77 ± 0.16	0.86 ± 0.15	0.4895
LV FS (%)	38.24 ± 6.98	44.07 ± 7.60	43.69 ± 5.92	0.0694
MV E/A ratio	0.93 ± 0.15	0.90 ± 0.19	0.89 ± 0.21	0.8185
TAPSE (cm)	0.44 ± 0.12	0.34 ± 0.10	0.41 ± 0.11	0.1907
LA volume (ml)	1.0 ± 0.29*	1.37 ± 0.86	1.89 ± 0.76*	0.0148
LA FS (%)	54.25 ± 11.27	49.59 ± 13.10	41.50 ± 13.11	0.0706
LARS (%)	32.79 ± 11.09*	24.35 ± 7.19	20.28 ± 4.67*	0.0405
LV GLS-AV (%)	–22.09 ± 3.39**†	–14.24 ± 4.62†	–11.95 ± 4.93**	<0.0001
RV LS-AV (%)	–19.55 ± 4.15	–17.38 ± 5.70	–15.42 ± 2.84	0.2674
Circumferential Strain (%)	–27.92 ± 5.97†	–18.62 ± 7.56†	–24.17 ± 2.62	0.0155

Data are presented as mean ± SD and analyzed by one-way analysis of variance with Tukey’s multiple comparison test. * p < 0.05, ** p < 0.01 when Group 1 vs Group 3; # p < 0.05, ## p < 0.01 when Group 2 vs Group 3; † p < 0.05, †† p < 0.01 when Group 1 vs Group 2. LA = left atrial; AO = aorta; IVSd = interventricular septal at end-diastole; LVPWd = left ventricular free proximal wall diameter at end-diastole; LVIDd = left ventricular internal diameter at end-diastole; IVSs = interventricular septal at end-systole; LVPWs = left ventricular free proximal wall diameter at end-systole; LVIDs = left ventricular internal diameter at end-systole; FS = fractional shortening; PV Vmax = pulmonary valve maximum blood velocity; AV Vmax = aortic valve maximum blood velocity; IVRT = isovolumic relaxation time; TAPSE = tricuspid annular plane systolic excursion; LARS = left atrial reservoir strain; LV GLS-AV = left ventricular global longitudinal strain–average; RV LS-AV = right ventricular longitudinal strain–average.

## DISCUSSION

Our findings confirm that distinct strain curve patterns correspond to different cardiomyopathic phenotypes, and the strain-volume approach extends concepts previously described in humans. In the hypertrophic phenotypes, strain reduction occurred without marked LV dilatation, resulting in curves closer to those of the normal controls. These observations support the concept that strain curve analysis captures the interplay between function and remodeling in feline cardiomyopathy. This study employed a multi-chamber approach, providing a more comprehensive assessment of myocardial and atrial function than prior studies that focused on individual chambers. The role of LA strain is particularly noteworthy. Conventional indices, such as the LA volume index, provide information on chronic remodeling but are less responsive to dynamic changes in diastolic function [16]. LARS may offer additional functional information beyond LA/AO, consistent with findings from human and canine studies, in which atrial strain may improve the assessment of diastolic dysfunction in felines [17].

### Global longitudinal strain

A previous study showed that LVGLS values in control and preclinical HCM cats are comparable to our findings [30]. Longitudinal strain (LS) mapping through a bull’s-eye plot enables the quantification of regional myocardial deformation and visual assessment of spatial heterogeneity in LV contractility (Figures 1 A–H). Results provide a more detailed evaluation of both the location and extent of myocardial dysfunction, enabling identification of regional abnormalities that may be missed by global indices such as FS. In the present analysis, the bull’s-eye map demonstrated segmental variations in LS, with reduced deformation predominantly within the septal and inferior regions, while the lateral and apical segments retained near-normal values. This finding of regional inhomogeneity of strain is particularly relevant in the context of HCM, where FS often remains normal or supranormal because of concentric remodeling and reduced cavity size. In these cases, FS or EF alone underestimates systolic dysfunction, as geometric alterations mask impaired myocardial shortening. In contrast, GLS and regional LS patterns can be used to sensitively detect subtle abnormalities in contractility.

The current strain pattern may indicate segmental hypertrophy with nonuniform wall thickening. Regional strain reduction has been associated with myofiber disarray and interstitial fibrosis, providing additional diagnostic insight beyond conventional echocardiographic measures. Therefore, incorporating LS bull’s-eye mapping into routine echocardiographic analysis may enhance the differentiation of hypertrophic phenotypes and may help to improve the recognition of early myocardial dysfunction in cats.

Some previous studies reported preserved longitudinal systolic strain and strain rate (SR), with only diastolic abnormalities in early and late diastolic SR (E and A SR), whereas others reported significant reductions in both the systolic and diastolic deformation indices. These discrepancies are likely attributable to differences in study design, sample size, degree of hypertrophy, and methods used for segmental and global strain analysis. In one earlier study, no change in longitudinal systolic strain (S) or the SR was observed, although a reduction in diastolic SR was documented [25]. That cohort consisted of untreated cats with relatively mild hypertrophy and limited heterogeneity in disease severity, and segmental strain data were not provided. The relatively small sample size and uniformity of the study population likely limited its statistical power and generalizability. Conversely, another investigation demonstrated decreased circumferential and longitudinal systolic strain in asymptomatic cats with HCM compared with healthy controls, but these differences were less evident in cats showing mild clinical signs such as difficulty breathing and tachypnea [3]. However, subgroup analyses with few animals per category may have further reduced the robustness of their findings.

The results of the present study show that cats with asymptomatic HCM exhibit significantly reduced circumferential and longitudinal deformation when compared with the healthy control Group, despite preserved FS. These alterations suggest the presence of subclinical myocardial dysfunction, which is consistent with early contractile impairment detectable by STE before conventional echocardiographic indices decrease. Interestingly, our data revealed preserved circumferential strain in Group 3 (Table 2), in agreement with previous studies reporting that circumferential deformation may be maintained until more advanced stages of hypertrophy. One recent investigation demonstrated preserved endocardial circumferential strain but reduced epicardial strain in cats with dynamic left ventricular outflow tract obstruction, resulting in an increased epicardial-to-endocardial strain ratio [4–6]. These findings imply a layer-specific pattern of deformation, in which subendocardial fibers remain functional for longer, whereas subepicardial dysfunction may contribute to disease progression.

The circumferential and radial strain patterns observed in this study (Figures 2A and 2B) illustrate synchronous contraction in healthy cats and reduced strain amplitude with delayed peak timing in HCM cats, indicating heterogeneous regional deformation. These results emphasize the importance of incorporating layer-specific and regional strain analyses to fully characterize myocardial mechanics in feline HCM. Overall, circumferential strain assessment complements longitudinal measurements, providing a multidimensional understanding of myocardial dysfunction in feline cardiomyopathy. The integration of strain imaging across myocardial layers and orientations may enhance the early detection and staging of HCM, supporting improved risk stratification and therapeutic monitoring in clinical practice.

### LA strain

Previous studies in human cardiology have shown that both an increased LA volume index and a reduced LARS are independently associated with the LV mass index. However, a previous study stated that they are not always directly linked to myocardial fibrosis quantified by cardiac magnetic resonance imaging (CMR). In the present feline cohort, which included cats diagnosed with HCM, we observed similar trends: LARS correlated closely with conventional echocardiographic indices of LV diastolic dysfunction, particularly the mitral inflow E/A ratio and tricuspid regurgitation (TR) velocity (Figures 3A–F). Cats with lower LARS values demonstrated greater LV wall thickness, increased fibrosis extent, and reduced LV LS, indicating that impaired atrial compliance accompanies ventricular hypertrophy and stiffness. Notably, a reduced LARS-defined diastolic dysfunction grade was also associated with resting LV outflow tract obstruction, highlighting the interaction between abnormal diastolic filling and dynamic obstruction in feline HCM.

The relationship between LARS and other diastolic markers persisted even when adjusted for potential confounders such as arrhythmia or the risk of sudden cardiac death. However, no outcome data, such as congestive heart failure (CHF), arterial thromboembolism, or survival, were assessed in this study. These findings suggest that LARS may provide independent prognostic information beyond traditional echocardiographic parameters. The progressive reduction in LARS may therefore serve as an early marker of disease severity and a predictor of HCM-related morbidity, including hospitalization for CHF.

The incorporation of LARS into echocardiographic assessment provides a quantitative and reproducible tool for evaluating diastolic performance. In addition to LV and RV strain analyses, LARS adds substantial value in identifying early hemodynamic changes, stratifying the risk of affected cats, and guiding clinical management. As in human cardiology, this parameter represents an important advancement in the noninvasive evaluation of LV filling pressures and diastolic function in feline HCM. Collectively, these results reinforce the concept that strain-based imaging, particularly when combining LVGLS and LARS, enhances the diagnostic precision of echocardio-graphy and provides superior insight into the pathophysiology and progression of feline cardiomyopathy.

### Right ventricular longitudinal strain (RVLS)

RVLS, derived from STE, has emerged as a reliable and reproducible index of RV systolic function. It enables the detection of subtle functional changes that often precede alterations in conventional parameters such as fractional area change or tricuspid annular plane systolic excursion (TAPSE). In both human and veterinary cardiology, the RVLS provides additional prognostic information and has become an important complement to the standard echocardiographic assessment of biventricular function. In this study, the RVLS was quantified from the RV-focused apical four-chamber view (Figures 4A–D), focusing specifically on the free-wall segments. The strain–time curves demonstrated segmental variability, suggesting early RV involvement in HCM. Results emphasize that ventricular interdependence in feline HCM extends beyond the LV, as increased LV wall stiffness and diastolic dysfunction can impose secondary strain on RV performance through interventricular coupling and shared myocardial fibers. In human cardiology, an absolute RVLS value greater than -20% is generally considered normal, with less negative values indicating impaired systolic function [31]. Comparable strain ranges have been reported in healthy cats, although species-specific reference intervals remain under development [32]. Reduced RV strain has been associated with various pathological states, including pulmonary hypertension, congenital heart disease, cardiomyopathies, and systemic RV [33].

Human studies have shown that RV strain has independent prognostic significance in predicting adverse cardiac events, particularly in patients with cardiomyopathy or pulmonary hypertension [34]. RVLS may remain preserved in early-stage feline HCM due to the relatively low prevalence of secondary pulmonary hypertension and limited changes in right ventricular afterload. The incorporation of RVLS into feline echocardiographic protocols could enhance early recognition of right-sided dysfunction in conditions such as restrictive or HCM.

The integration of RVLS with LV and LA strain analyses provides a more comprehensive understanding of biventricular–atrial mechanics and allows for earlier detection of global myocardial dysfunction. This approach, using strain echocardiography, may improve disease staging and monitoring of therapeutic outcomes in cats with cardiomyopathy [35], thereby establishing strain echocardiography as a valuable adjunctive marker in routine cardiac evaluation. Clinical application of strain echocardiography in cats is growing. Our results extend these findings by integrating volumetric analysis to provide a comprehensive assessment of cardiac mechanics that may aid in early diagnosis, prognosis, and treatment monitoring.

### Limitations

A limitation of the present study is that it was conducted at a single center with a relatively small sample size. Some cats were excluded todue of suboptimal image quality and elevated heart rates, reflecting the technical challenges associated with feline patients. Additionally, threshold values for feline LALS remain to be standardized across platforms and populations. Larger, multicenter studies are warranted to validate diagnostic cut-offs and prognostic implications. Another limitation of this study is that circulating cardiac biomarkers, such as N-terminal pro-B-type natriuretic peptide (NT-proBNP), were not evaluated. Because myocardial deformation parameters and cardiac biomarkers may reflect different pathophysiological processes, combining STE with biomarker assessment may improve the detection of early myocardial dysfunction in cats.

## CONCLUSION

This study demonstrates that strain echocardiography provides valuable additional information for assessing myocardial function in cats with HCM phenotypes. Key findings include significantly reduced LVGLS and LARS in cats with hypertrophic phenotype and LA enlargement compared with healthy controls, while RVLS remained relatively preserved. Strain–volume curve analysis revealed impaired myocardial deformation patterns, characterized by flatter, right-shifted curves in affected cats, despite preserved conventional systolic indices such as FS. These results highlight the complementary value of multi-chamber strain parameters in detecting subclinical dysfunction and differentiating hypertrophic phenotypes.

The integration of LVGLS, RVLS, and LARS into routine echocardiographic evaluation can improve early detection of myocardial dysfunction, enhance phenotypic characterization, and support more accurate disease staging in feline HCM. This approach may assist clinicians in identifying cats at higher risk of progression and guide therapeutic decisions, ultimately contributing to better management of this common cardiac disease.

The study employed a comprehensive multi-chamber strain analysis combined with strain–volume curve evaluation, providing a more complete assessment of cardiac mechanics than previous investigations focused on single chambers. The use of standardized imaging protocols and offline analysis with vendor-specific software ensured high reproducibility.

The study was conducted at a single center with a relatively small sample size. Some cats were excluded due to suboptimal image quality and elevated heart rates, reflecting the technical challenges associated with feline patients. Additionally, threshold values for feline LALS remain to be standardized across platforms and populations. Circulating cardiac biomarkers, such as NT-proBNP, were not evaluated.

Larger, multicenter studies are warranted to validate diagnostic cut-offs and prognostic implications of these strain parameters. Future research should incorporate longitudinal follow-up to assess the predictive value of strain indices for clinical outcomes and to evaluate the combined use of STE with cardiac biomarkers to improve detection of early myocardial dysfunction in cats.

In conclusion, multi-chamber strain echocardiography integrating LVGLS, RVLS, and LARS, together with strain–volume curve analysis, offers a sensitive and comprehensive tool for evaluating myocardial mechanics in cats with HCM. This approach enhances diagnostic precision beyond conventional echocardiography and holds promise for improving early detection, risk stratification, and clinical management of feline cardiomyopathy.

## DATA AVAILABILITY

The data generated during the study are included in the manuscript.

## GENERATIVE AI DECLARATION

The authors declare that generative artificial intelligence (AI) tools were used solely to improve language, grammar, and readability during manuscript preparation. All scientific content, data analysis, interpretation of results, and conclusions were developed and verified by the authors. The authors take full responsibility for the accuracy, integrity, and originality of the work presented, and no AI tool was listed as an author.

## AUTHORS’ CONTRIBUTIONS

SP: Conceptualized and designed the study, performed the data analysis, and drafted the manuscript. YXF and CP: Analyzed the results and revised and edited the manuscript. All authors have read and approved the final version of the manuscript.
